# Psychopathia Sexualis

**Published:** 1893-09

**Authors:** 


### BOOK NOTICES.

Psychopathia Sexualis, with Especial Reference to Contrary Sexual Instinct. A Medico-Legal Study. By Dr. R. von Krafft-Ebing, Professor of Psychiatry and Neurology. University of Vienna. Authorized translation of the seventh, enlarged and revised, German edition. By Charles Gilbert Chaddock, M. D., Professor of Nervous and Mental Diseases, Marion-Sims College of Medicine, St. Louis; Fellow of the Chicago Academy of Medicine; Corresponding Member of the Detroit Academy of Medicine;

Associate Member of the American Medico-Psychological Association, etc. In one royal octavo volume, 436 pages, extra cloth, $3.00 net; sheep, $4.00 net. Sold only by subscription. Philadelphia: The F. A. Davis Co., Publishers, 1914 and 1916 Cherry Street.

This very remarkable book, one of the most remarkable from the standpoint of the subject treated ever issued from the press, gives the reader a glimpse of some of the underlying causes of many of the most remarkable social phenomena which are forever occurring and forever puzzling the uninitiated. Crimes, we call them, crimes of the most revolting type, compromising alike the innocent and the guilty, shattering family unions, occupying the attention of the courts, and filling the newspapers with the unsavory recitals.

The influences at work are more powerful for evil than even the love of money. The results even more criminal than those of money-getting.

The object of the book, as stated in the preface, is "a description of the pathological manifestations of the sexual life, and an attempt to refer them to their underlying conditions."

In these pages will be found a revelation of wickedness that must make the reader feel that he has had a peep through the "gates ajar" of Hades.

Yet no prurient imagination can find food for gratification in the perusal of this work. Arranged upon a perfectly scientific basis, it can gratify no idle curiosity, inflame no latent lust. Wherever the descriptions pass the bounds of propriety the worst passages are obscured by being rendered in Latin. The work can be utilized by the physician only. For him it throws a flood of light upon the phenomena which he witnesses in his daily life, which make his task difficult, and which involve him in one way or another, hampering his progress, and adding to his anxieties. To quote again from the preface: "He who makes the psychopathology of sexual life the object of scientific study sees himself placed on a dark side of human life and misery, in the shadows of which the god-like creations of the poet become hideous masks, and morals and æsthetics seem out of place in the image of God."

"For the physician himself, sexual anomalies, treated as they are in a distant manner in text-books on psychiatry, are in greater part a *terra incognita.* Exact knowledge of the causes and conditions of development of sexual aberrations, and of the influence on them of hereditary constitution, education, the impressions of everyday life, and modern refined civilization is the prerequisite for a rational prophylaxis of sexual aberrations, and for a correct sexual education. Without a careful study of the circumstances which attend the *development* of sexual anomalies, we should never be in a position to use effectual therapeusis. The majority of these unfortunates—Krafft-Ebing calls them Nature's stepchildren—are devoid of insight into their malady: like insane patients, destitute of understanding of the ethical development of man, they are happy in their abnormal instinctive tendency. For this reason, in spite of the great prevalence of uranism, very few of its subjects seek medical treatment."

This lengthy quotation from the preface will serve better than any words of the reviewer to show the object of the book, its scope, and its great value as an addition to medical literature.

